# Insulin Receptor Substrate-2 Regulates the Secretion of Growth Factors in Response to Amino Acid Deprivation

**DOI:** 10.3390/ijms26020841

**Published:** 2025-01-20

**Authors:** Ayaka Takahashi, Haruka Furuta, Hiroki Nishi, Hiroyasu Kamei, Shin-Ichiro Takahashi, Fumihiko Hakuno

**Affiliations:** 1 Department of Animal Resource Sciences, Graduate School of Agricultural and Life Sciences, The University of Tokyo, Tokyo 113-8657, Japan; ayk006100@gmail.com (A.T.); harukafuruta0507@gmail.com (H.F.); shwest@mail.saitama-u.ac.jp (H.N.); atkshin@g.ecc.u-tokyo.ac.jp (S.-I.T.); 2 Area of Regulatory Biology, Division of Life Science, Graduate School of Science and Engineering, Saitama University, Saitama 338-8570, Japan; 3 Faculty of Biological Science and Technology, Institute of Science and Engineering, Kanazawa University, Kanazawa 920-1192, Japan; hkamei@se.kanazawa-u.ac.jp

**Keywords:** amino acid deprivation, IRS-2, VEGF-D, stress response

## Abstract

Insulin receptor substrates (IRSs) are well-known mediators of the insulin and insulin-like growth factor (IGF)-I signaling pathways. We previously reported that the protein levels of IRS-2, a molecular species of IRS, were upregulated in the livers of rats fed a protein-restricted diet. This study aimed to elucidate the physiological role of IRS-2, whose level increases in response to protein restriction in cultured hepatocyte models. Hepatocyte-derived cell lines subjected to amino acid deprivation showed increased *IRS2* mRNA and IRS-2 protein levels due to increased *IRS2* transcription and translation, respectively. Amino acid deprivation markedly increased vascular endothelial growth factor-D (VEGF-D) secretion. Remarkably, the amino acid deprivation-induced VEGF-D secretion was suppressed by IRS-2 knockdown and enhanced by IRS-2 overexpression. These results suggest that IRS-2 is an intercellular signaling molecule that extracellularly transmits information on amino acid deprivation stress by regulating the secretion of growth factors such as VEGF-D. Moreover, this function of IRS-2 is distinct from its currently accepted function as a mediator of the insulin/IGF-I signaling pathways. This study demonstrates that IRS-2 can modulate protein secretion in an insulin-independent manner and greatly expands our understanding of the role of IRS-2, which is upregulated in response to amino acid deprivation.

## 1. Introduction

Insulin receptor substrates (IRSs) are well-known mediators of the insulin or insulin-like growth factor (IGF)-I signaling pathways. IRSs are tyrosine-phosphorylated by insulin/IGF-I receptor tyrosine kinases activated by insulin or IGF-I [1]. Tyrosine-phosphorylated IRSs activate the downstream phosphatidylinositol-3 kinase (PI-3K) and mitogen-activated protein kinase (MAPK) pathways, resulting in various physiological activities, including cell proliferation, differentiation, and survival [2,3]. IRS-knockout (KO) animals exhibit growth retardation and diabetic symptoms, including insulin resistance and obesity [4,5,6,7,8,9], suggesting that IRSs are essential for the biological activities of insulin/IGF-I. Moreover, IRS-1 KO mice exhibit growth retardation and their peripheral tissues become insulin-resistant or have reduced insulin sensitivity; however, the condition does not progress to diabetes because of a compensatory increase in insulin secretion [4,10]. In contrast, IRS-2 KO mice exhibit insulin resistance in target tissues, such as the muscles and liver, and reduced pancreatic insulin secretion, leading to type 2 diabetes and obesity [7,8,9,11,12,13]. However, it has been reported that IRS-2 plays an important role in regulating growth but is not essential for blood glucose-lowering effects in rats [14].

We previously reported that the protein level of IRS-2, a molecular species of IRS, increased in the liver of rats fed a protein-deficient diet [15]. In these rats, insulin sensitivity was enhanced, gluconeogenic activity was reduced, and a fatty liver was formed [15,16]. Initially, it was expected that the increased expression of IRS-2, a mediator of insulin signaling, would induce hepatic lipid accumulation and decrease the expression of gluconeogenic rate-limiting enzymes by enhancing insulin signaling activity. However, subsequent analyses have revealed that enhanced insulin signaling activation may not directly cause hepatic lipid accumulation or decreased gluconeogenic activity in cultured hepatocyte models [17,18]. Furthermore, the physiological role of increased IRS-2 protein levels induced by amino acid deprivation remains unknown. In addition to low amino acid diets, other nutrients in the diet or some diseases are known to be responsible for decreased blood amino acid levels. Recently, decreased blood amino acid levels have been reported in rats fed a high-fat diet [19] and in humans with amnestic mild cognitive impairment [20]. Thus, this study was undertaken to evaluate unknown functions of IRS-2, which increases under amino acid deprivation.

Amino acid deprivation not only acts as a regulator of general translational repression but also implies a deficiency of synthetic materials for protein synthesis and can be considered as translational repression from both points of action. Under amino acid deprivation, general control nonderepressible 2 (GCN2), a serine/threonine kinase activated by an increase in the amount of free tRNA or stalled ribosomes, phosphorylates eukaryotic translation initiation factor-2α (eIF2α) subunits, resulting in the inhibition of general translation subunits [21,22]. In contrast, activating transcription factor 4 (ATF4) induces gene expression in response to amino acid deprivation stress by promoting translation through selective regulation [23,24,25]. Moreover, the activation of the GCN2-ATF4 pathway selectively induces the expression of several proteins that play important roles under amino acid deprivation stress conditions.

Recently, we showed that IRS-2 promotes the secretion of matrix metalloproteinase-9 (MMP-9) in the PC3 cell line, a malignant prostate cancer cell line expressing high levels of IRS-2 [26]. Based on these data, we hypothesized that IRS-2 plays an unknown role in promoting growth factor and/or cytokine secretion. In this study, we found that amino acid deprivation upregulated *IRS2* transcription and translation in rat primary hepatocytes and HuH-7 hepatoma cells and that this increased expression of IRS-2 promoted the secretion of growth factors and cytokines, such as vascular endothelial growth factor-D (VEGF-D), in an insulin-independent manner via a novel mechanism. These phenomena can be nutritional stress responses that transmit information on nutritional status, such as hormones in the blood, to the entire body.

## 2. Results

### 2.1. Amino Acid Deprivation Upregulates Both IRS2 Transcription and Translation in Hepatocyte-Derived Cells

**Figure 1:**
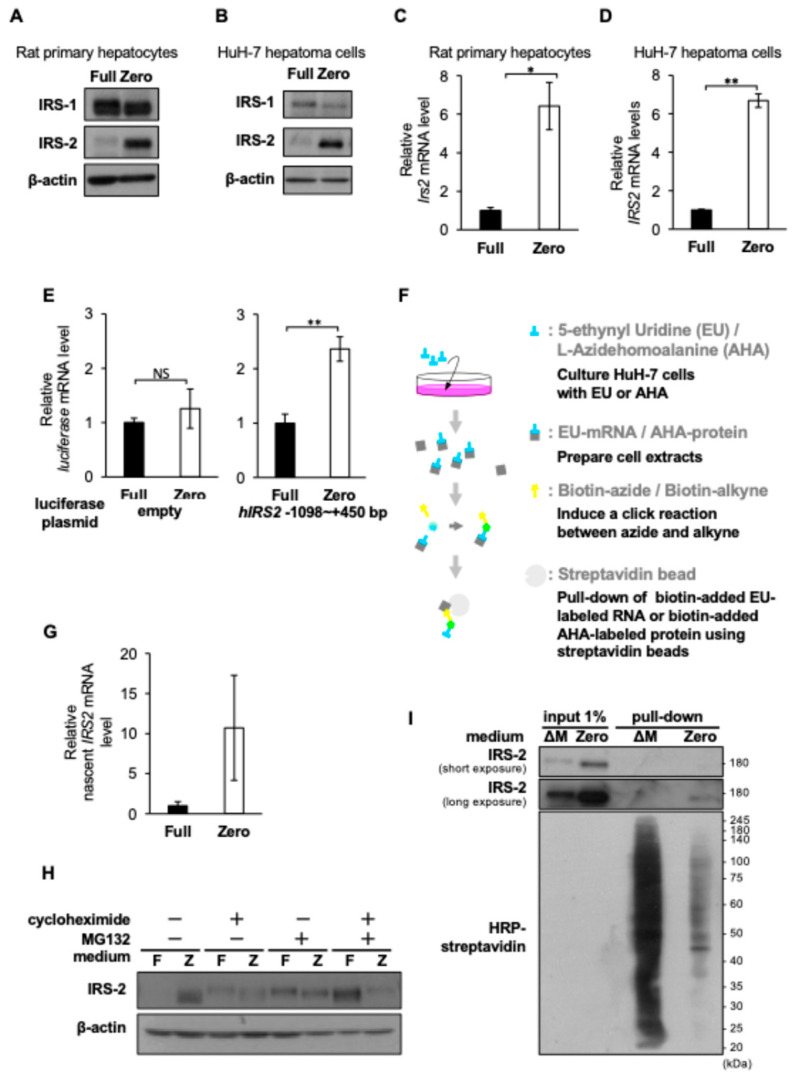
Effects of amino acid deprivation on *IRS2* mRNA levels and IRS-2 protein levels in hepatocyte-derived cells. Rat primary hepatocytes (**A**) and HuH-7 human hepatoma cells (**B**) were cultured for 24 h in a medium containing 20 amino acids (Full) or in a medium with no amino acids (Zero). Rat primary hepatocytes (**C**) and HuH-7 human hepatoma cells (**D**) were cultured for 12 h in the Full or Zero medium. (**E**) HuH-7 cells were transfected with firefly luciferase reporter plasmids (empty or *hIRS2* containing −1098/+450 bp of the *human IRS2* gene promoter region). Then 39 h after transfection, the spent media were changed and cells were cultured for an additional 9 h. (**F**) Method for extracting newly synthesized mRNA or protein from cells. (**G**) HuH-7 cells were cultured in Full or Zero medium with 0.5 µM 5-ethynyluridine for 6 h. (**C**–**E**,**G**) *IRS2* mRNA levels (**C**,**D**,**G**) or firefly luciferase mRNA levels (**E**) were measured using real-time qPCR. (**H**) HuH-7 cells were cultured in Full or Zero medium with or without 10 µg/mL cycloheximide or 10 µM MG132 for 24 h. (**I**) HuH-7 cells were cultured in methionine-depleted medium (ΔM medium) or Zero medium for 14 h. Next, 50 µM L-azidehomoalanine (AHA) was added to the medium and the cells were cultured for an additional 4 h. (**A**,**B**,**G**,**H**) Cell lysates were subjected to immunoblotting. In (**C**–**F**), *IRS2* mRNA levels (**C**,**D**,**F**) or firefly luciferase mRNA levels (**E**) were normalized against *ACTB* mRNA levels. Bar graphs are presented as the fold changes of the columns on the far left. Bar: mean ± S.E.M. NS not significant, * p < 0.05, ** *p* < 0.005, Student’s *t*-test. (**C**–**E**): *n* = 3, (**G**): *n* = 6. The experiments were performed multiple times independently and a representative result was presented.

Our previous study showed that dietary protein deprivation increased IRS-2 protein levels in the rat liver [15]. To determine whether this phenomenon observed in rats could be recapitulated in cell culture, hepatocyte-derived cells were cultured in amino acid-modified media. For these experiments, we prepared serum-free amino acid-sufficient medium (Full) and serum-free amino acid-depleted medium (Zero) (Table 1). When rat primary hepatocytes and HuH-7 human hepatoma cells were cultured in the Zero medium, IRS-2 protein levels increased compared to those cultured in the Full medium (Figure 1A,B). In addition, *IRS2* mRNA levels were higher in cells grown in the Full medium than those in the Zero medium (Figure 1C,D).

Next, we assessed the effect of amino acid deprivation on *IRS2* transcription in HuH-7 cells. First, we constructed a *human IRS2* promoter (−1098 to +450 upstream of the *human IRS2* gene)-driven luciferase reporter (Figure 1E). After transfection with a luciferase reporter plasmid, HuH-7 cells were cultured in the Full or Zero medium and firefly luciferase mRNA levels were measured. Promoter activity increased in cells grown in the Zero medium compared with those in the Full medium. Next, the levels of newly synthesized *IRS2* mRNA were measured using Nascent RNA capture methods based on the click reaction (Figure 1F). As shown in Figure 1G, newly synthesized *IRS2* mRNA levels increased in the Zero medium (Figure 1G), indicating that amino acid deprivation increased *IRS2* mRNA levels by enhancing transcription. *IRS2* mRNA levels and promoter activity were partially decreased by ATF4 knockdown (Figure S1A–C) or treatment with the GCN2-ATF4 pathway inhibitor ISRIB (Figure S1D–F).

Proteins are in a state of dynamic equilibrium, in which protein synthesis and degradation occur continuously in the cell. Therefore, we examined the contribution of proteasomal degradation and translation to the increase in IRS-2 protein levels in response to amino acid deprivation. IRS-2 protein levels in cells grown in the Zero medium decreased upon the addition of the protein synthesis inhibitor cycloheximide (Figure 1H). IRS-2 protein levels in cells grown in the Full medium increased following treatment with the proteasome inhibitor MG132 (Figure 1H). These results suggest that IRS-2 degradation was enhanced in the Full medium, but IRS-2 synthesis was enhanced in the Zero medium. Although the quantity and variety of nascent proteins possibly decreased in the amino acid-deprived medium, newly synthesized IRS-2 protein levels increased in the Zero medium (Figure 1I), indicating that amino acid deprivation increased IRS-2 protein levels by enhancing both transcription and translation.

### 2.2. Amino Acid Deprivation Activates the PI 3-Kinase Pathway in an IRS-2-Dependent Manner

**Figure 2:**
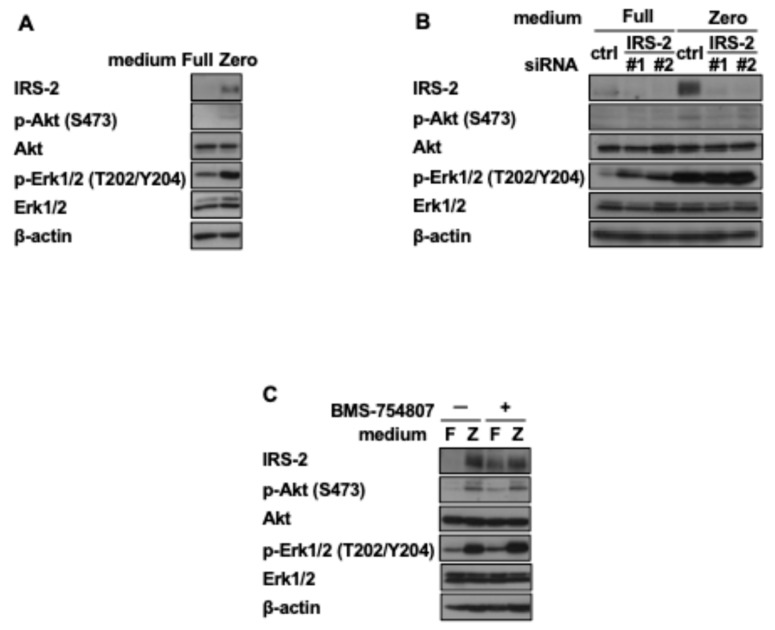
Effects of amino acid deprivation and increase in IRS-2 protein levels on the insulin signaling pathway. (**A**) HuH-7 cells were cultured in the Full or Zero medium for 24 h. (**B**) HuH-7 cells were transfected with control siRNA or siRNA against IRS-2. Then, 24 h after transfection, the spent medium was changed to Full or Zero medium and the cells were cultured for an additional 24 h. (**C**) HuH-7 cells were cultured in Full or Zero medium with or without 1 nM BMS-754807 for 24 h. (**A**–**C**) The cell lysates were subjected to immunoblotting. The experiments were performed multiple times independently and a representative result was presented.

IRS-2 is a well-known mediator of the insulin signaling pathway. Hence, we then examined whether amino acid deprivation activated insulin signaling pathways. HuH-7 cells were cultured in Full or Zero medium for 24 h and then we examined the phosphorylation levels of Akt, a downstream kinase of the PI-3K pathway, and Erk1/2, a downstream kinase of the MAPK pathway. Amino acid deprivation increased the phosphorylation of Akt and Erk1/2 upon insulin stimulation (Figure 2A). Notably, Akt phosphorylation was suppressed by IRS-2 knockdown but not by the insulin/IGF-I receptor kinase inhibitor BMS-754807 (Figure 2B,C). In contrast, Erk1/2 phosphorylation was not suppressed upon IRS-2 knockdown (Figure 2B). These results indicate that PI-3K pathway activation in the Zero medium occurred in an IRS-2-dependent manner, but MAPK pathway activation did not occur through IRS-2.

### 2.3. IRS-2 Promotes VEGF-D Secretion in an Insulin-Independent Manner

**Figure 3:**
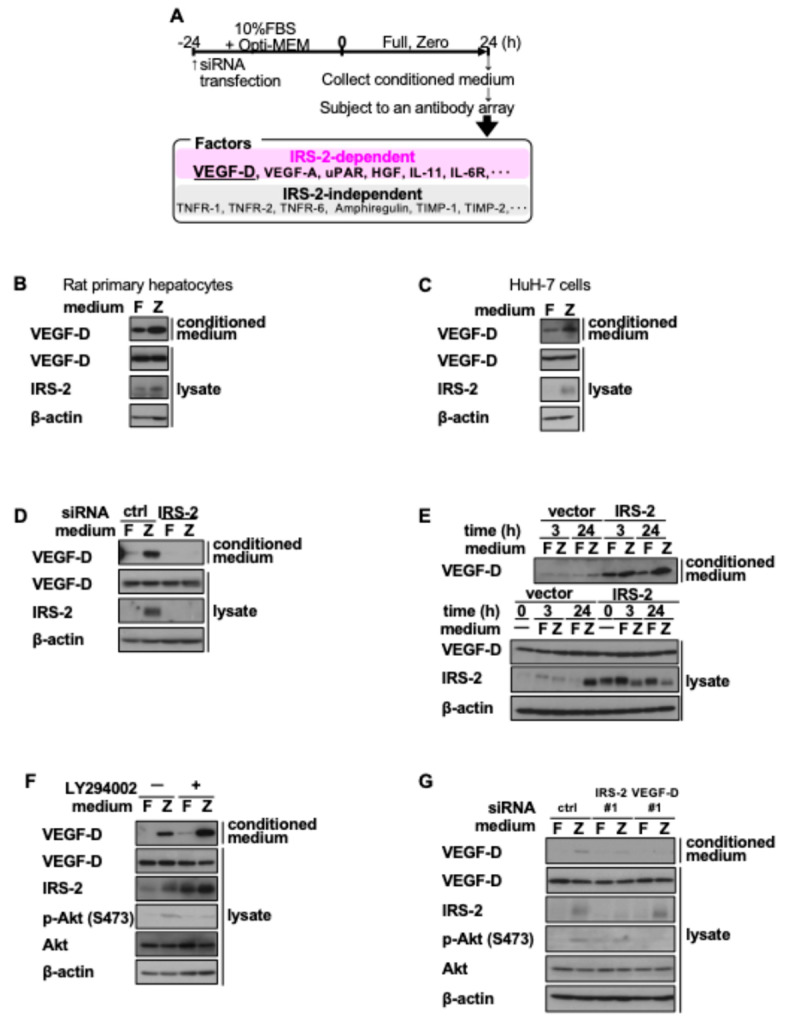
Effects of amino acid deprivation and increase in IRS-2 protein levels on VEGF-D secretion. (**A**) HuH-7 cells were transfected with control siRNA or siRNA against IRS-2. Then 24 h after transfection, media were changed to Full or Zero medium and the cells were cultured for an additional 24 h. The conditioned media were collected and subjected to an antibody array. Rat primary hepatocytes were cultured for 6 h in Full or Zero medium (**B**) and HuH-7 human hepatoma cells were cultured for 24 h in Full or Zero medium (**C**). (**D**) HuH-7 cells were transfected with control siRNA or siRNA against IRS-2. Then 24 h after transfection, the spent media was changed to Full or Zero medium and cells were cultured for an additional 24 h. (**E**) HuH-7 cells stably expressing IRS-2 or vector were cultured in Full or Zero medium for 0, 3, or 24 h. (**F**) HuH-7 cells were cultured in Full or Zero medium with or without 10 µM LY294002 for 24 h. (**G**) HuH-7 cells were transfected with control siRNA, siRNA against IRS-2, or VEGF-D. Then 24 h after transfection, the spent media was changed to Full or Zero medium and the cells were cultured for an additional 24 h. (**B**–**G**) The cell lysates and their conditioned medium were subjected to immunoblotting. The experiments were performed multiple times independently and a representative result was presented.

We found that the PI-3K pathway was activated in response to amino acid deprivation without insulin stimulation. These results led us to develop the working hypothesis that the PI-3K pathway is activated by ligands secreted by HuH-7 cells in response to amino acid deprivation. Therefore, we investigated whether amino acid deprivation affects the levels of secretory factors. We cultured HuH-7 cells in Full or Zero medium for 24 h and examined the secretory factor levels in the conditioned medium using antibody arrays (Figure 3A). Our screening analyses identified various growth factors, including vascular endothelial growth factor-D (VEGF-D), as potential candidates. HuH-7 cells or primary hepatocytes were cultured in Full or Zero medium for 24 h and the conditioned medium was prepared. The conditioned medium was then subjected to sodium dodecyl sulfate-polyacrylamide gel electrophoresis (SDS-PAGE), followed by immunoblotting with an anti-VEGF-D antibody. VEGF-D secretion was markedly increased when cells were cultured in the Zero medium compared to those in the Full medium (Figure 3B,C). IRS-2 knockdown suppressed Zero medium-induced VEGF-D secretion (Figure 3D), while IRS-2 overexpression enhanced VEGF-D secretion (Figure 3E). Conversely, VEGF-D secretion was not suppressed by the PI-3K pathway inhibitor LY294002 (Figure 3F), indicating that IRS-2 upregulated VEGF-D secretion in a PI-3K activation-independent manner. In addition, VEGF-D knockdown suppressed Akt phosphorylation in cells cultured in the Zero medium without affecting IRS-2 expression levels (Figure 3G), suggesting that VEGF-D secretion by IRS-2 is required for Akt phosphorylation in cells cultured in the Zero medium.

### 2.4. IRS-2 Promotes Microvesicle (MV) Secretion, Including VEGF-D

**Figure 4:**
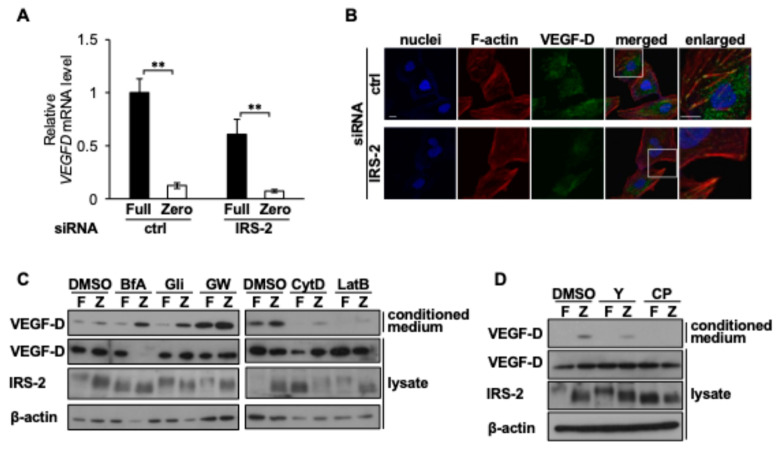
Mechanisms of IRS-2-induced VEGF-D secretion. (**A**) HuH-7 cells were transfected with control siRNA (ctrl) or siRNA against IRS-2. Then 24 h after transfection, the spent media were changed to Full or Zero medium and cells were cultured for an additional 12 h. (**B**) HuH-7 cells were seeded on coverslips and transfected with scramble (ctrl) siRNA or IRS-2 siRNA. Then 24 h after transfection, media were changed to Zero medium and the cells were cultured for an additional 6 h. Cells were stained with Hoechst33342 (blue), phalloidin (red), and anti-VEGF-D antibody (green). Enlarged images of the white boxes were shown on the right. White arrows indicate the colocalization of VEGF-D with F-actin. Scale bar: 10 µm. (**C**) HuH-7 cells were cultured in Full or Zero medium with or without 5 µg/mL bafilomycin A1 (BafA), 50 µM glibenclamide (Gli), 10 µM GW4869 (GW), 10 µM cytochalasin D (CytD), or 10 µM latrunculin B for 6 h. (**D**) HuH-7 cells were cultured in Full or Zero medium with or without 10 µM Y-27632 (Y) or 100 µM calpeptin (CP) for 6 h. (**C**,**D**) The cell lysates and their conditioned medium were subjected to immunoblotting. Bar: mean ± S.E.M., ** *p* < 0.005, Student’s *t*-test, *n* = 3. The experiments were performed multiple times independently and a representative result was presented.

Next, we examined how IRS-2 increases extracellular VEGF-D protein levels. *VEGFD* mRNA levels decreased when cells were cultured in Zero medium and remained unchanged upon IRS-2 knockdown (Figure 4A), suggesting that the reduced extracellular VEGF-D protein levels in IRS-2 knockdown cells were not due to a decrease in the amount of VEGF-D mRNA. Immunostaining analysis using an anti-VEGF-D antibody revealed that VEGF-D partially colocalized with F-actin in HuH-7 cells (Figure 4B, upper panel, white arrows) and this colocalization disappeared in IRS-2 knockdown cells (Figure 4B, lower panel). Since VEGF-D secretion may be regulated by extracellular vesicles (EVs), we treated cells with various chemicals that block or enhance secretory pathways. Notably, treatment with GW4869, which is known to inhibit exosome secretion but increase microvesicle (MV) secretion [27,28], significantly increased VEGF-D secretion (Figure 4C). In contrast, VEGF-D secretion decreased after treatment with Y-27632 or calpeptin, which inhibited MV secretion at different points [28] (Figure 4D).

## 3. Discussion

In the present study, we found that IRS-2 protein levels increased in response to amino acid deprivation in both rat primary hepatocytes and HuH-7 human hepatoma cells. It is widely accepted that cells under stress conditions, including nutrient starvation, reduce their cellular load by inhibiting translation. However, even under amino acid deprivation, several proteins are selectively translated via the GCN2-ATF4 pathway. *IRS2* mRNA levels and the promoter activity of *IRS2* were partially decreased by ATF4 knockdown or treatment with a GCN2-ATF4 pathway inhibitor, suggesting that *IRS2* transcription is, at least in part, promoted by the activation of the GCN2-ATF4 pathway. Translation inhibitors decreased IRS-2 protein levels and levels of newly synthesized IRS-2 protein increased under amino acid deprivation. Therefore, both the transcription and translation of *IRS2* were promoted under amino acid deprivation. These data strongly suggest that IRS-2 might have critical roles in the stress response, as the protein levels of IRS-2 increased during amino acid deficiency when the environment was not conducive to protein synthesis.

Furthermore, the PI-3K pathway, which is downstream of the insulin signaling pathway, is activated in response to amino acid deprivation in an IRS-2-dependent manner. Analysis of secreted proteins in the cell culture supernatants revealed a marked increase in VEGF-D levels under amino acid deprivation. Moreover, VEGF-D knockdown attenuated the activation of the PI-3K pathway in a manner similar to IRS-2 knockdown. These data suggest that VEGF-D induces the activation of the PI-3-kinase pathway in response to amino acid deficiency. We also found that VEGF-D secretion was decreased by IRS-2 knockdown and increased by IRS-2 overexpression. In contrast, PI-3K pathway inhibitors did not decrease VEGF-D secretion, strongly suggesting that VEGF-D secretion is independent of insulin signaling activation. This function of IRS-2 to activate insulin signaling through the secretion of VEGF-D is distinct from its currently accepted function as a mediator of insulin signaling pathways.

VEGF is a member of the platelet-derived growth factor family and cystine-knot superfamily of growth factors [29,30,31,32]. The mammalian VEGF family consists of VEGF-A to -F [33] and placental growth factor (PIGF). They bind to three receptors with different affinities and specificities: VEGFR-1, VEGFR-2, and VEGFR-3 [34]. VEGFs regulate multiple signaling pathways involved in cell growth, proliferation, and survival and are known to be important for angiogenesis during embryonic development. VEGF-D has physiological activities, mainly in VEGFR-2-mediated angiogenesis and VEGFR-3-mediated lymphangiogenesis [35,36,37]. In the livers of VEGF-D KO mice, the expression levels of genes associated with lipid metabolism were downregulated and inflammation were upregulated [38], suggesting that VEGF-D has some functions involved in fatty liver formation and subsequent development of cirrhosis and hepatocarcinoma following inflammation and fibrosis. Taken together with our data showing that protein malnutrition induces fatty liver formation [38], this raises the possibility that the secretion of VEGF-D by IRS-2 in response to amino acid deprivation might affect liver inflammation. It is well established that VEGF-D induces biological effects not only on VEGF-secreting cells themselves in an autocrine/paracrine manner, but also on other types of cells and other tissues in an endocrine manner. Thus, increased IRS-2 levels in hepatocytes in response to amino acid deprivation may play a novel physiological role in triggering biological responses through VEGF-D secretion. Therefore, the liver and systemic phenotypes of VEGF-D should be carefully studied in the future, as the bioactivity of VEGF-D may differ between species [39,40].

Previous studies have shown that IRS-2 protein levels increased in the liver of rats fed a protein-deficient diet [15,16]. In these previous studies case, IRS-2 protein levels in the skeletal muscle and white adipose tissue were unchanged compared to those in the controls. Thus, the increase in IRS-2 protein levels in response to reduced dietary amino acid levels was liver-specific. The liver is an important organ for monitoring the nutritional status of an organism because amino acids and other nutrient molecules absorbed in the gastrointestinal tract enter the body directly via the portal vein. Hence, liver IRS-2, whose level increases in response to a decrease in ingested amino acid levels, may be a part of the amino acid monitoring system in vivo. In HuH-7 cells, IRS-2 regulates the secretion of EVs, including VEGF-D, which appear as MVs. We have found that IRS induces the expression of diverse bioactivities through tyrosine phosphorylation-independent interactions with IRS-associated proteins (IRSAPs) [41]. IRSAPs that have been isolated by screening included those involved in secretion. As IRS-2 has been found to be involved in EV secretion in this study, it is highly likely that various EV secretions, including VEGF-D, are regulated through IRSAPs. EVs such as MVs act as messengers during intercellular communication, implying that EVs secreted by a cell interact with a receptor cell and cause changes in its physiological functions [42]. EVs exchange various components, from nucleic acids to lipids to proteins, between cells and serve as signaling mediators in normal cellular homeostatic processes or as a consequence of pathological developments [42,43,44]. Hence, IRS-2 may be an important stress effector that regulates signaling to the surrounding cells and cells in other tissues by modulating the secretion of VEGF-D and other substances, thereby inducing a hepatic or systemic stress response or homeostasis. Moreover, we have previously reported that IRS-2 promotes MMP-9 secretion in the prostate cancer cell line PC3 and induces cancer cell overgrowth and malignant transformation. In this paper, we have successfully shown for the first time that IRS-2 may promote the secretion of factors important for normal cell proliferation, survival and stress responses.

In this study, the contribution of the GCN2-ATF4 pathway to the increase in *IRS2* mRNA levels is likely to be partial. Further investigation of other transcription factors that induce *IRS2* mRNA up-regulation in response to amino acid deprivation may provide additional insight into amino acid deprivation signaling in cells, tissues, and organisms. In addition, the physiological roles of VEGF-D, which is secreted in response to increase in IRS-2 protein levels during amino acid deprivation, and its potential effects mediated through PI 3-kinase pathway activation require further investigation. Clarification of the role of liver-derived VEGF-D in liver tissue cells or cells from other tissues may provide evidence for the non-canonical function of IRS-2 as a factor responsible for intercellular communication through secretion regulation of molecules such as VEGF-D. There were also limitations in the methods used to isolate, identify, and assess the size and purity of the EVs. In addition, the specific stage at which IRS-2 regulates EV secretion remains unclear. Based on these considerations, further investigation is needed to determine whether IRS-2 affects the secretion of EVs, particularly MVs.

In conclusion, this study showed that IRS-2 can modulate protein secretion in an insulin-independent manner, which significantly expands our understanding of the role of IRS-2, which is upregulated in response to amino acid deprivation. Elucidation of this detailed mechanism will help us better understand the regulation of systemic stress response and disease by controlling autocrine, paracrine, and endocrine responses.

## 4. Materials and Methods

### 4.1. Materials

William’s E medium (#W4125), 10× Earle’s buffered salt solution (EBSS), 100× MEM vitamin solution, and fetal bovine serum (FBS) were purchased from Sigma-Aldrich (St. Louis, MO, USA). Dulbecco’s modified Eagle’s medium (DMEM), phosphate-buffered saline, and Hanks’ buffered saline solution were purchased from Nissui Pharmaceutical Co. (Tokyo, Japan). All other reagents used in this study were commercially available.

### 4.2. Cell Culture and Cell Experiments

Rat primary hepatocytes were prepared as previously described [17] and cultured in William’s E medium supplemented with 10% FBS and antibiotics. Briefly, parenchymal hepatocytes were isolated from 8-week-old male Wistar rats by perfusing the liver with collagenase in a perfusion buffer [8 g/L NaCl, 0.4 g/L KCl, 0.56 g/L CaCl_2_, 0.078 g/L NaH_2_PO_4_·2H_2_O, 0.151 g/L Na_2_HPO_4_·12H_2_O, 2.38 g/L HEPES, 0.006 g/L Phenol red, 350 mg/L Collagenase Type X (Wako, Osaka, Japan), 150 mg/L, Trypsin inhibitor from soybean (#T9003, Sigma Aldrich), 130 mg/L Trypsin inhibitor from chicken egg white (#T9253, Sigma Aldrich), 0.35 g/L NaHCO_3_, pH 7.4]. Rats were purchased from Charles River Laboratories (Kanagawa, Japan). HuH-7 cells (human hepatoma cell line, JCRB0403, Japanese Collection of Research Bioresources (JCRB) Cell Bank, Osaka, Japan) were grown in DMEM supplemented with 10% FBS and antibiotics. All the cells were cultured in 5% CO_2_ at 37 °C.

When all cells reached 80% confluency, the medium was changed to the experimental medium (Table 1) and the cells were cultured for the indicated durations. In this study, serum-free media containing sufficient amino acids and containing no amino acids are referred to as the Full medium and Zero medium, respectively [17]. The experiment was independently conducted twice and the reproducibility of the results was confirmed.

All rat care and experiments conformed to the Guidelines for Animal Experiments of the University of Tokyo and were approved by the Animal Research Committee of the University of Tokyo.

### 4.3. Plasmid Preparation

Human genomic DNA was extracted from HuH-7 cells, and the DNA segment around the transcription initiation site of *IRS2* was amplified via PCR. The primer sequences used in this study were: *human IRS2* −317~+450 bp Forward, 5′-CATCTCCTCCGCCGGCATCCACAACAAGCCGCTG-3′ and *human IRS2* −317~+450 bp Reverse, 5′-CCGGCGGAGGAGATGATCCTCGAGGCTAGCGAGCT-3′. The amplified DNA fragment was inserted into the pGL4.11[luc2] vector using an In-Fusion HD Cloning Kit (Takara Bio, Kyoto, Japan) for the luciferase reporter assay. The initiation codon in the luciferase gene of the vector was eliminated because the *IRS2* promoter fragment already contains an initiation codon.

pFLAG-IRS-2 was constructed as described previously [45]. The plasmids expressing *human IRS2* were cloned into pFLAG-4-5.

### 4.4. siRNAs

siRNAs used in this study were obtained from Nippon Gene Materials Co. (Tokyo, Japan). The siRNAs comprised the following sequences: IRS-2 #1, 5′-UCGGCUUCGUGAAGCUCAA-3′; IRS-2 #2, 5′-GGCUGAGCCUCAUGGAGCA-3′; ATF4#1, 5′-GCGUCAAUGUGCUUGUACA-3′; ATF4#2, 5′-CUGCUUACGUUGCCAUGAU-3′; ATF4#3, 5′-GCCACUAGGUACCGCCAGA-3′; and VEGF-D#1, 5′-GGGCUCCAGUAAUGAACAU-3′.

### 4.5. Transfection with Plasmids or siRNAs

The luciferase reporter assay plasmids were transfected into HuH-7 cells using polyethyleneimine as described below. The overexpression plasmids were transfected into HuH-7 cells using Lipofectamine 3000 Transfection Reagent (Thermo Fisher Scientific, Waltham, MA, USA) via forward transfection method, following the manufacturer’s protocol. The siRNAs were transfected into HuH-7 cells using Lipofectamine RNAiMAX Transfection Reagent (Thermo Fisher Scientific) via reverse transfection, following the manufacturer’s protocol.

### 4.6. RNA Extraction and Real-Time Quantitative PCR (qPCR)

Total RNA was extracted from liver tissues or cells using TRIzol Reagent (Invitrogen, Carlsbad, CA, USA) and cDNA synthesis was performed using ReverTra Ace qPCR RT Master Mix (Toyobo, Osaka, Japan). cDNA was subjected to qPCR using a THUNDERBIRD Next SYBR qPCR Mix (Toyobo) and an Applied Biosystems StepOne Real-Time PCR System (Thermo Fisher Scientific). *ACTB* mRNA was measured as an internal control for all the samples and used for data normalization. The primer sequences used in this experiment were: *human IRS2* Forward, 5′-AGCTTCTTCTTCATCGAGGTG-3′; *human IRS2* Reverse, 5′-AACTCGAAGAGCTCCTTGAG-3′; *rat Irs2* Forward, 5′-AGCTTCTTCTTCATCGAGGTG-3′; *rat Irs2* Reverse, 5′-AACTCGAAGAGCTCCTTGAG-3′; *human ATF4* Forward, 5′-TTCTCCAGCGACAAGGCTAAGG-3′; *human ATF4* Reverse, 5′-CTCCAACATCCAATCTGTCCCG-3′; *human VEGFD* Forward, 5′-GACTGGAAGCTGTGGAGATGCA-3′; *human VEGFD* Reverse, 5′-GGCTGCACTGAGTTCTTTGCCA-3′; *human ACTB* Forward 5′-TTCCTTCCTGGGCATGGAG-3′, *human ACTB* Reverse, 5′-GCAGTGATCTCCTTCTGCATC-3′; *rat Actb* Forward, 5′-CTAAGGCCAACCGTGAAAAGAT-3′; and *rat Actb* Reverse 5′-TCCAGGCTGTGTTGTCCCT-3′.

### 4.7. Luciferase Reporter Assay

Promoter activity was measured using a firefly luciferase reporter plasmid. Briefly, HuH-7 cells were seeded in 12-well plates at a density of 4 × 10^5^ cells/well and incubated overnight. The cells were then transfected with luciferase reporter plasmids (empty or *hIRS2* −1098/+450 bp [*human IRS2* promoter region]) using polyethyleneimine. After 39 h, the spent medium was changed to the experimental medium and the cells were cultured for another 9 h. After the extraction of the total RNA, cDNA synthesis and qPCR were performed to detect the level of luciferase mRNA.

### 4.8. Nascent mRNA Analysis

Nascent RNA was isolated using a Click-iT^®^ Nascent RNA Capture Kit (Life Technologies, Carlsbad, CA, USA) according to the product’s instructions. Next, cDNA synthesis and qPCR were performed to detect the levels of nascent *IRS2* mRNA. Briefly, HuH-7 cells were cultured for 6 h in Full or Zero medium supplemented with 0.2 mM 5-ethynyluridine (EU). After total RNA extraction, EU-labeled RNA and biotin azide were cycloadducted with azide and alkyne, a click reaction, and pulled down using streptavidin beads. The amount of EU-labeled *IRS2* mRNA was measured using real-time qPCR.

### 4.9. Preparation of Conditioned Medium

After the cells were cultured in the experimental medium for the indicated durations, the medium was collected, centrifuged to remove cell debris, and concentrated 20-fold using centrifugal filters (pore size: 3 kDa; Merck Millipore Ltd., TullaGreen, Carrigtwohill, Cork, Ireland).

### 4.10. Immunoblotting

Cell lysates and conditioned media were prepared using standard methods and subjected to SDS-PAGE. Gel-separated proteins were transferred onto polyvinylidene fluoride membranes (Merck Millipore, Billerica, MA, USA) and protein bands were visualized using a Western Lightning Plus-ECL Enhanced Chemiluminescence Substrate (PerkinElmer, Waltham, MA, USA). The following antibodies were used: anti-IRS-1 antibody (Sigma-Aldrich, St. Louis, MO, USA, #06-248), anti-IRS-2 antibody (Santa Cruz Biotechnology, Santa Cruz, CA, USA, #sc-390761), anti-ATF4 antibody (Cell Signaling Technology, Inc. [CST], Danvers, MA, USA, #11815), anti-Akt antibody (CST, #9272), anti-phospho-Akt antibody (CST, #9271), anti-Erk1/2 antibody (CST, #9102), anti-phospho-Erk antibody (CST, #9101), anti-VEGF-D antibody (Abcam, Cambridge, UK, #ab155288), anti-β-actin antibody (Sigma-Aldrich, #A2228), HRP-conjugated streptavidin (Thermo Fisher Scientific, #N100), HRP-conjugated anti-mouse IgG (GE Healthcare, Chicago, IL, USA, #NA931V), and HRP-conjugated anti-rabbit IgG (GE Healthcare, #NA934V) (Table 2).

### 4.11. Nascent Protein Measurement and Analysis

Nascent protein was labeled with Click-iT^®^ AHA (L-azidehomoalanine) (Life Technologies) and captured with a Click-iT^®^ Protein Reaction Buffer Kit (Life Technologies) according to the product’s instructions. Next, immunoblotting was conducted to detect the level of nascent IRS-2 protein using an anti-IRS-2 antibody and nascent total protein HRP-streptavidin.

### 4.12. Antibody Array

Semi-quantitative detection of 120 human cytokines and chemokines in the supernatants was performed using a RayBio C-series Human Cytokine Antibody Array C1000 (RayBiotech, Inc., Norcross, GA, USA; #AAH-CYT-1000-2) according to the manufacturer’s instructions. Dot detection was performed using Western Lightning Plus-ECL Enhanced Chemiluminescence Substrate (PerkinElmer). Quantification of the dots was performed using ImageJ 1.53d software (National Institutes of Health (NIH), Bethesda, MD, USA). Raw numerical densitometry data were extracted and subjected to background subtraction before normalizing the signal of each cytokine against the positive control signal in each cytokine array.

### 4.13. Quantification and Statistical Analysis

Data are expressed as the mean ± standard error of the mean. Comparisons between two groups were performed using the Student’s *t*-test. Comparisons between more than two groups were performed using one- or two-way analysis of variance (ANOVA). If the *p*-value obtained from the ANOVA was less than 0.05, post-hoc tests indicated in each figure legend were performed. A value of *p* < 0.05 was considered statistically significant. All statistical calculations were performed using JMP^®^ Pro16 software (SAS Institute Inc., Cary, NC, USA). All statistical details regarding the experiments can be found in the corresponding figure legends.

## Figures and Tables

**Table 1:** Experimental media composition (mg/L).

	Full	Zero
Glycine	30.0	0
L-Alanine	35.6	0
L-Serine	42.0	0
L-Threonine	95.0	0
L-Cystine	48.0	0
L-Methionine	30.0	0
L-Glutamine	584.0	0
L-Asparagine·H_2_O	60.0	0
L-Glutamic acid	58.8	0
L-Aspartic acid	53.2	0
L-Valine	94.0	0
L-Leucine	105.0	0
L-Isoleucine	105.0	0
L-Phenylalanine	66.0	0
L-Tyrosine	72.4	0
L-Tryptophan	16.0	0
L-Lysine·HCl	146.0	0
L-Arginine·HCl	84.0	0
L-Histidine	31.0	0
L-Proline	46.0	0
EBSS	10%	10%
vitamin solution	1%	1%
NaHCO_3_	2.2	2.2
D-glucose	4.5	4.5

**Table 2:** Antibody list.

Antibody	Species	Company	Concentration
Anti-IRS-1 #06-248	Rabbit	Sigma-Aldrich	1:1000
Anti-IRS-2 (B-5): sc-390761	Mouse	Santa Cruz Biotechnology	1:200
Anti-ATF4 (D4B8) #11815	Rabbit	Cell Signaling Technology	1:1000
Anti-Akt #9272	Rabbit	Cell Signaling Technology	1:1000
Anti-phospho-Akt (Ser473) #9271	Rabbit	Cell Signaling Technology	1:1000
Anti-p42/44MAPK (Erk1/2) #9102	Rabbit	Cell Signaling Technology	1:1000
Anti-phospho-p42/44MAPK (Erk1/2) (Thr202/Tyr204) #9101	Rabbit	Cell Signaling Technology	1:1000
Anti-VEGFD [EPR8457] (ab155288)	Rabbit	Abcam	1:1000
Anti-β-actin #A2228	Mouse	Sigma-Aldrich	1:20,000
HRP-conjugated streptavidin #N100	Goat	Thermo Fisher Scientific	1:1000
HRP-conjugated anti-mouse IgG #NA931V	Goat	GE Healthcare	1:10,000
HRP-conjugated anti-rabbit IgG #NA934V	Goat	GE Healthcare	1:10,000

## Data Availability

The original contributions of this study are included in the article and Supplementary Materials. Further inquiries can be directed to the corresponding authors.

## Supplementary Materials

The following supporting information can be downloaded at: https://www.mdpi.com/article/10.3390/ijms26020841/s1.
