# Supplementary material for: Insulin Receptor Substrate-2 Regulates the Secretion of Growth Factors in Response to Amino Acid Deprivation
Source: Int J Mol Sci. 2025 Jan 20;26(2):841. doi: 10.3390/ijms26020841 (PMC11766276; doi:10.3390/ijms26020841)
Supplement: Supplementary file 1 [file ijms-26-00841-s001.zip › ijms-3424075-supplementary.pdf]

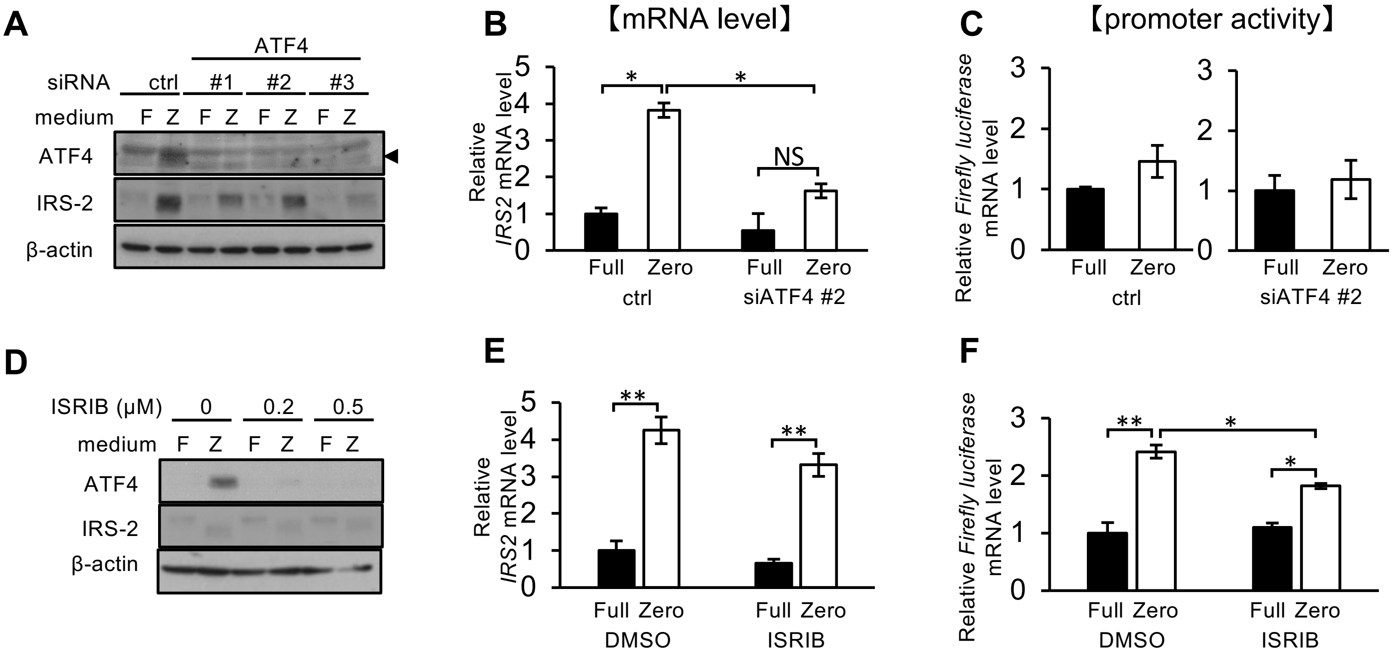

Figure S1. Amino acid deprivation upregulated *IRS2* transcription via activating GCN2-ATF4 pathway. (A) HuH-7 cells were transfected with control siRNA or siRNA against ATF4. 24 hours after transfection, medium were changed to Full or Zero medium and cells were cultured for additional 24 hours, proteins were analyzed by immunoblotting. (B) HuH-7 cells were transfected with control siRNA or siRNA against ATF4. 24 hours after transfection, medium were changed to Full or Zero medium and cells were cultured for additional 12 hours. (C) HuH-7 cells were transfected with control siRNA or siRNA against ATF4. 6 hours after transfection, HuH-7 cells were transfected with the Firefly luciferase reporter plasmids. 39 hours after transfection, medium were changed to Full or Zero medium and cells were cultured for additional 9 hours. (D) HuH-7 cells were cultured in Full or Zero medium with or without 0.2, 0.5 μM ISRIB for 6 hours. HuH-7 cells were cultured in Full or Zero medium with or without 0.5 μM ISRIB for 12 hours. And mRNA level (E) and promoter activity (F) were measured. In (B, C, E, F), *IRS2* mRNA levels and Firefly luciferase mRNA levels were normalized against *ACTB* mRNA levels. Bar graphs are presented as fold change of columns on the far left. Bar: mean ± S.E.M., $*p < 0.05$, Student's t-test, A: $n = 5$, B, C: $n = 3$. [means±SEM (n=3), $*p<0.05$, $**p<0.005$, (C) Student-T test, (B), (E), (F) Tukey-Kramer test].
